# Supplementary material for: Pro-inflammatory cytokines mediate the epithelial-to-mesenchymal-like transition of pediatric posterior fossa ependymoma
Source: Nat Commun. 2022 Jul 8;13:3936. doi: 10.1038/s41467-022-31683-9 (PMC9270322; doi:10.1038/s41467-022-31683-9)
Supplement: Supplementary file 1 — Supplementary Information [file 41467_2022_31683_MOESM1_ESM.pdf]

SUPPLEMENTARY INFORMATION

Pro-Inflammatory Cytokines Mediate the Epithelial-to-Mesenchymal-Like Transition of Pediatric Posterior Fossa Ependymoma

Rachael G. Aubin[*,1], Emma C. Troisi[*,1], Javier Montelongo[1], Adam N. Alghalith[1], MacLean P. Nasrallah[2], Mariarita Santi[2,3], and Pablo G. Camara[#]

[1] Department of Genetics and Institute for Biomedical Informatics,

[2] Department of Pathology and Laboratory Medicine,

Perelman School of Medicine, University of Pennsylvania,

Philadelphia, PA 19104, USA

[3] Department of Pathology, Children's Hospital of Philadelphia,

Philadelphia, PA 19104, USA

* These authors contributed equally to this work.

[#] Correspondence to: pcamara@pennmedicine.upenn.edu

Supplementary Figures

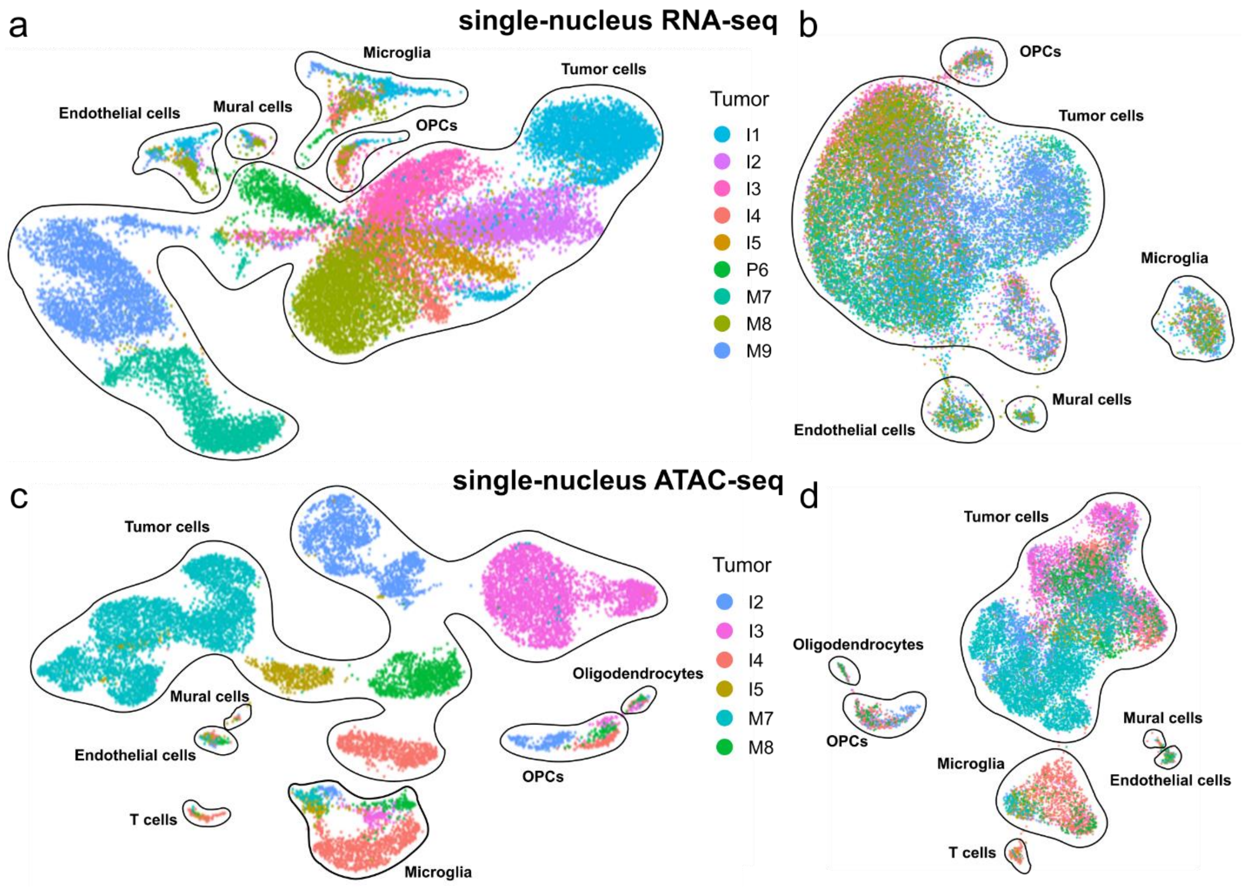

Supplementary Figure 1. UMAP representations of the uncorrected (a, c) and batch-corrected (b, d) single-nucleus RNA-seq (a, b) and ATAC-seq data (c, d). The representations are colored by the sample of origin.

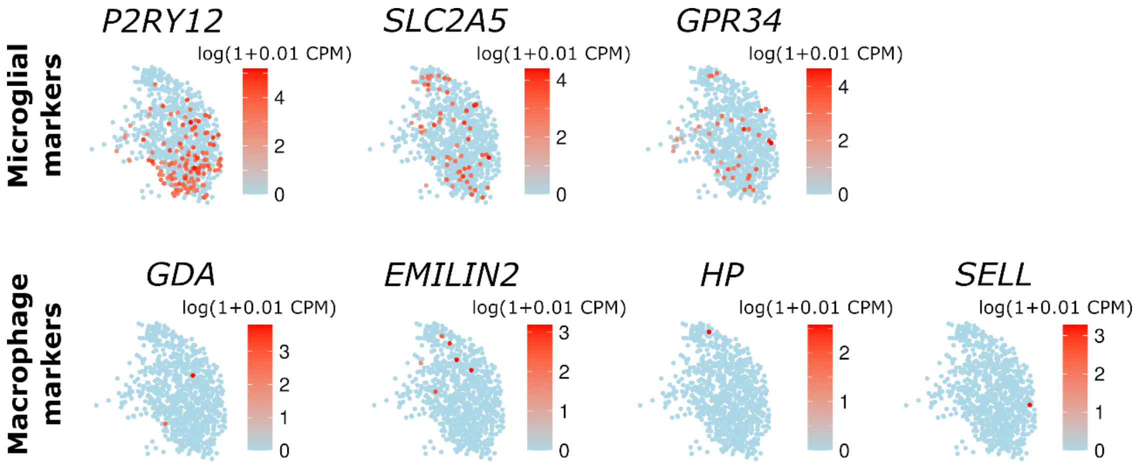

Supplementary Figure 2. Expression of resident microglia-specific and bone-marrow-derived macrophages marker genes in the microglia cluster.

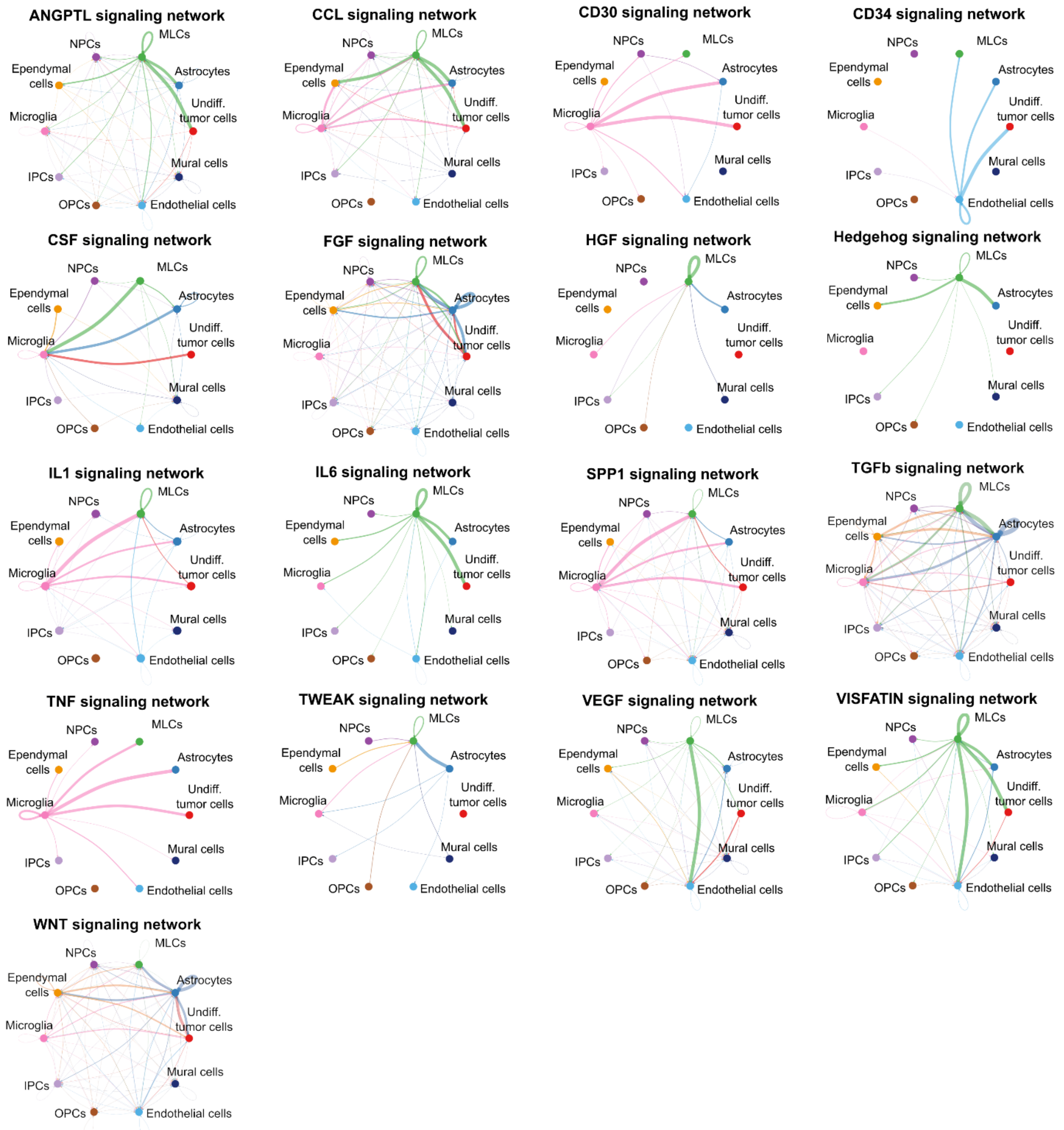

Supplementary Figure 3. Cytokine, chemokine, and growth factor cell-to-cell signaling networks associated with microglia and tumor cell populations. CellChat circle plots composed of differentially expressed genes coding for ligand and receptor/co-receptor pairs and categorized into signaling pathways. Edge widths represent the interaction strength of all ligand-receptor pairs within a signaling pathway between two cell populations. Edge colors indicate the cell population that sends the ligand. A complete list of inferred paracrine interactions and their

p-values can be found in Supplementary Table 3. NPCs: neural progenitor cells, IPCs: induced pluripotent stem cells, OPCs: oligodendrocyte precursor cells.

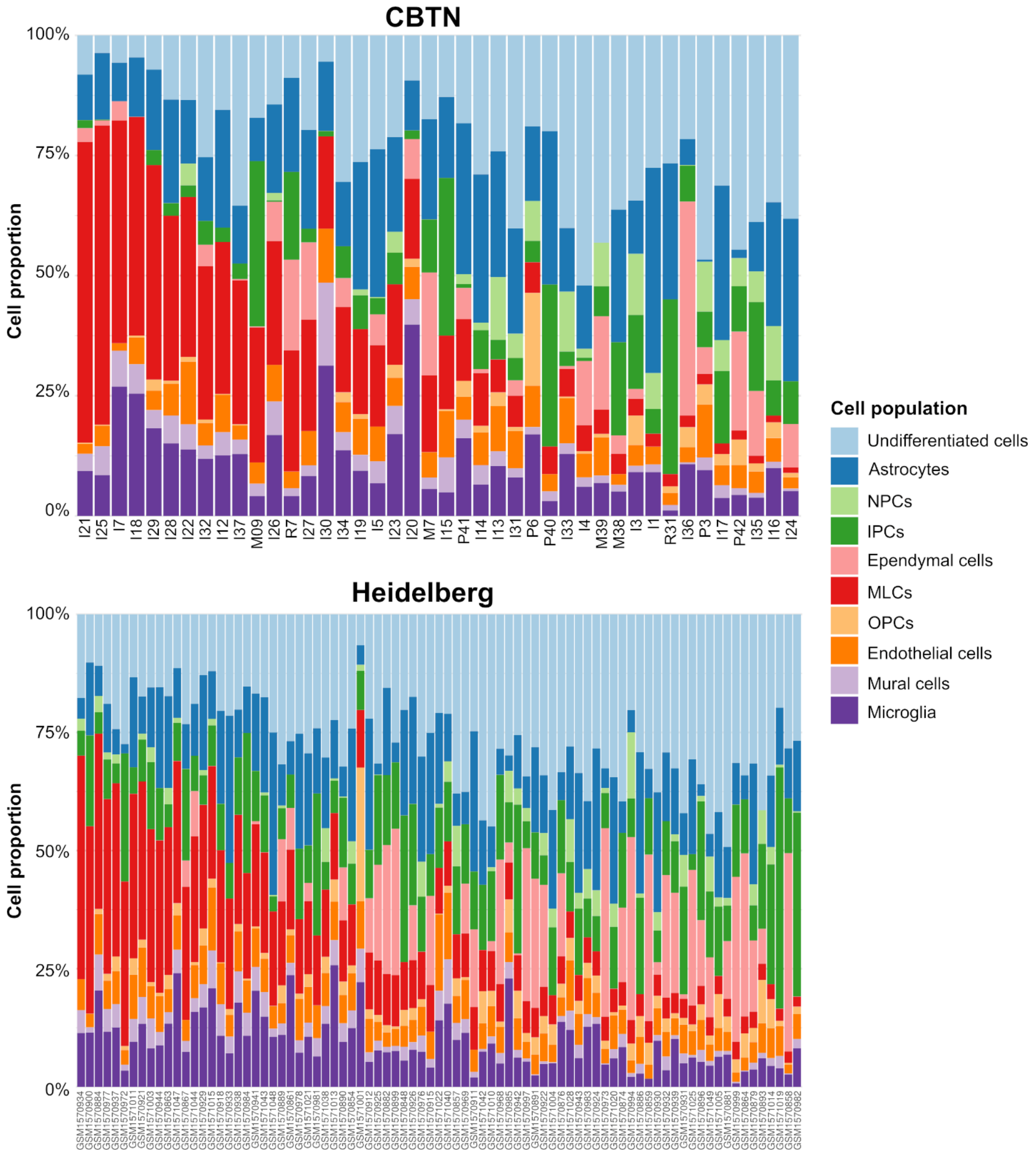

Supplementary Figure 4. Inferred cell population abundances in the CBTN and Heildeberg cohorts based on the bulk gene expression data of each tumor.

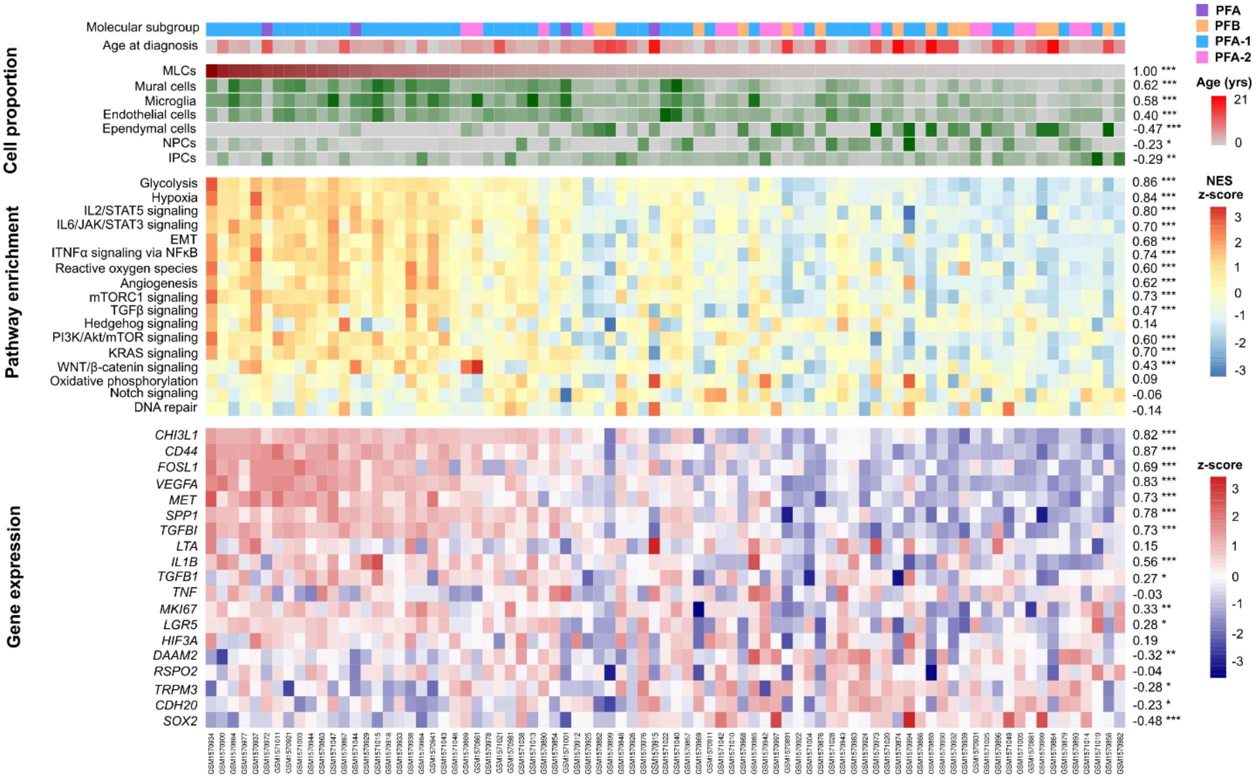

Supplementary Figure 5. The MLC population of posterior fossa ependymoma is associated with hypoxia, angiogenesis, glycolysis, and NFκB signaling gene expression programs. The same analysis as in Fig. 2C is performed for an independent cohort of 83 pediatric posterior fossa tumors from the Heildelberg dataset (Pajtler et al. 2015).

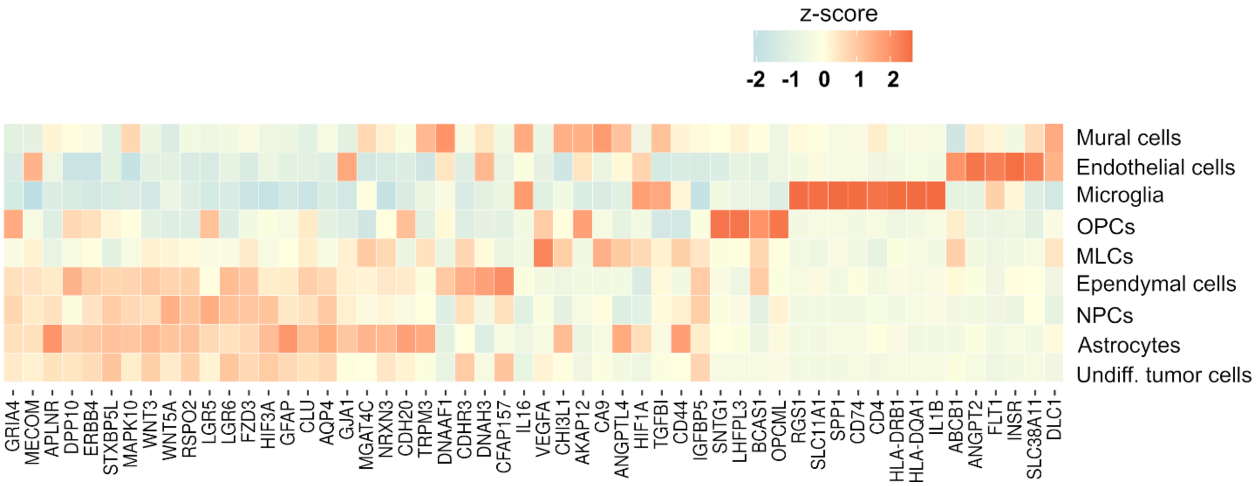

Supplementary Figure 6. Gene activity scores inferred from the single-nucleus ATAC-seq data for differentially expressed marker genes identified in the analysis of the single-nucleus RNA-seq data.

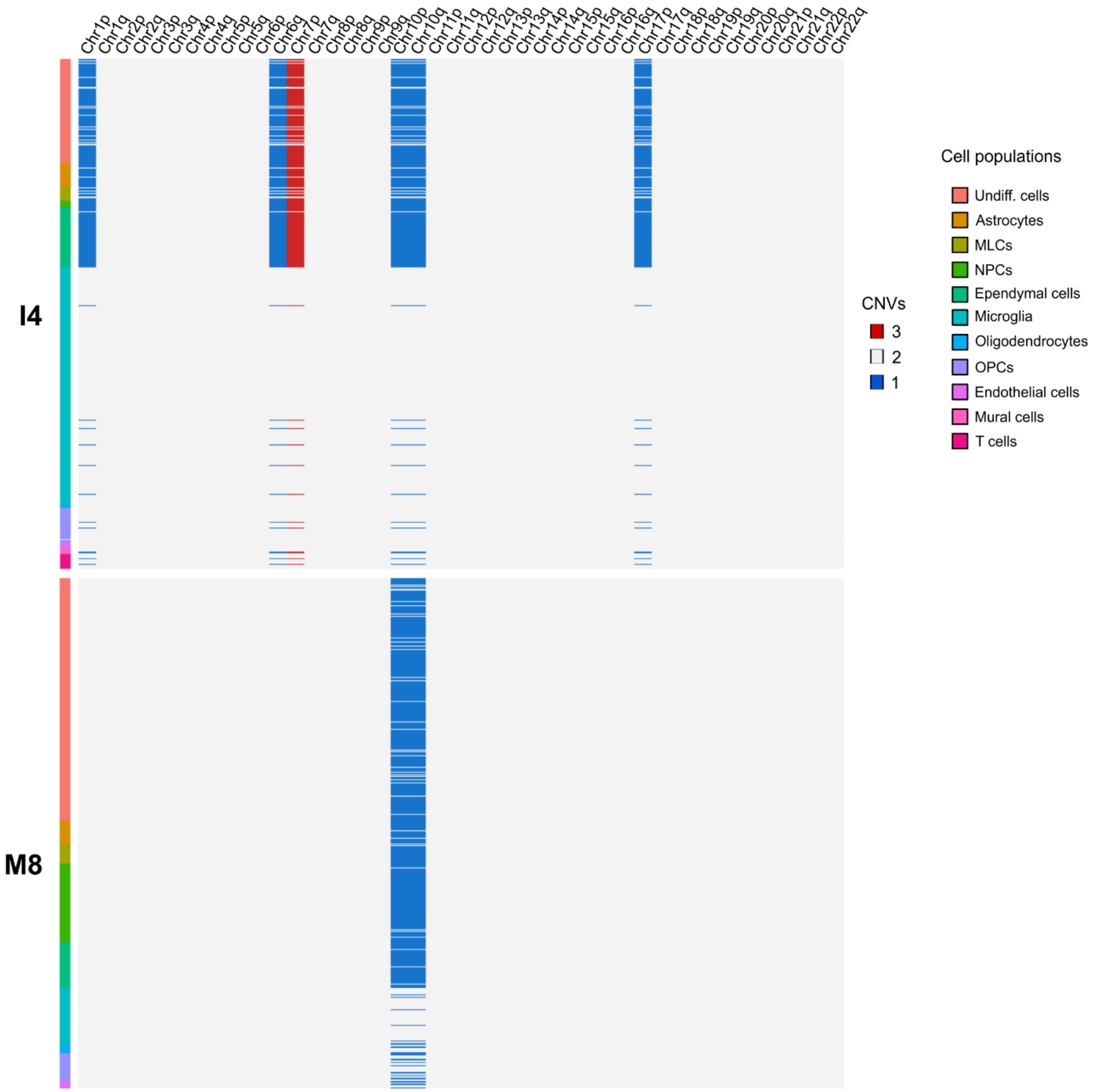

Supplementary Figure 7. Large-scale chromosomal aberrations inferred from the single-nucleus ATAC-seq data. Samples for which chromosomal aberrations were identified (I4 and M8) are shown. Rows correspond to cells and are ordered by cell population. Each column corresponds to a chromosome arm.

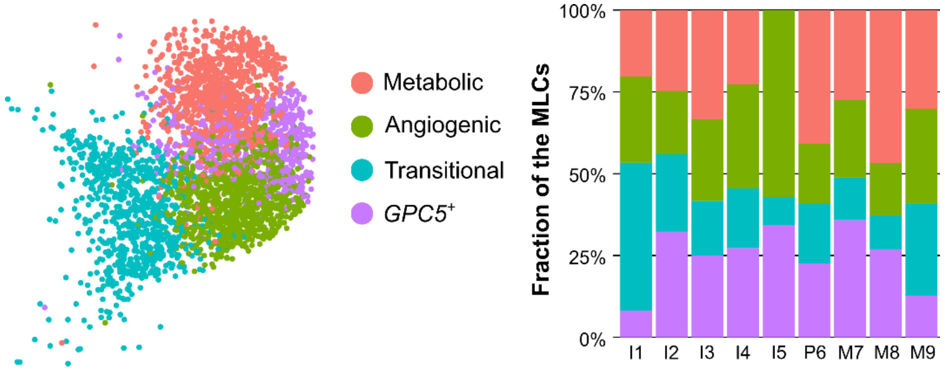

Supplementary Figure 8. Clustering analysis of the MLC population. MLCs were clustered into 4 distinct cell populations based on their gene expression profile. Left: the portion of the single-nucleus RNA-seq UMAP corresponding to the MLC population is colored by the resulting MLC subpopulations. These subpopulations were annotated based on the gene ontology enrichment analysis of differentially expressed genes (Supplementary Table 9). Right: the fraction of MLCs belonging to each subpopulation is shown for each tumor.

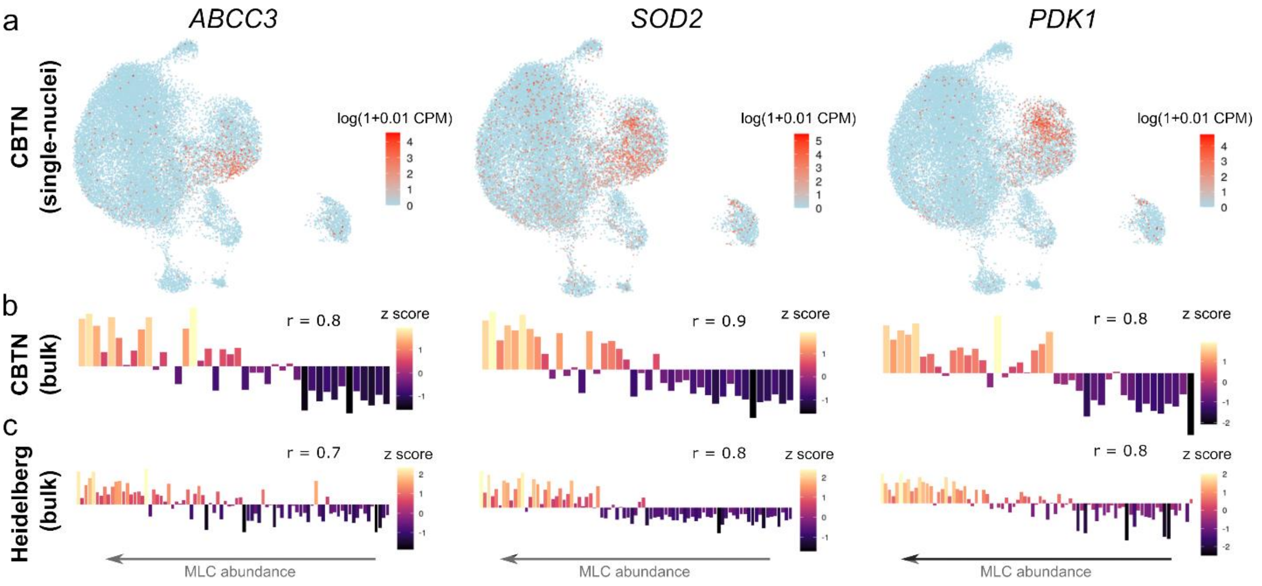

Supplementary Figure 9. Expression of multi-drug and radiation resistance genes in the MLC population. a) UMAP representation of the single-cell RNA-seq data showing high gene expression levels of *ABCC3*, *SOD2*, and *PDK1* in the MLC population. **b, c)** Barplots showing the expression level of these genes in each tumor of the CBTN (b) and Heidelberg (c) cohorts profiled for gene expression at the bulk level. Samples are ordered from left to right by decreasing order of inferred MLC abundance. The Spearman's correlation coefficient (*r*) between the gene expression level and the abundance of MLCs is shown in each case. Consistently with the results from single-nucleus RNA-seq data, samples with a high abundance of MLCs have high expression levels of these genes. All correlations were significant (2-sided test of association *p*-value $= 10^{-10}$).
