# Supplementary material for: Pro-inflammatory cytokines mediate the epithelial-to-mesenchymal-like transition of pediatric posterior fossa ependymoma
Source: Nat Commun. 2022 Jul 8;13:3936. doi: 10.1038/s41467-022-31683-9 (PMC9270322; doi:10.1038/s41467-022-31683-9)
Supplement: Supplementary file 3 — Description of Additional Supplementary Files [file 41467_2022_31683_MOESM3_ESM.pdf]

Description of Additional Supplementary Files

File Name: Supplementary Data 1
Description: **Summary information of the samples considered in the study. PFA**-1/2: Posterior fossa ependymoma group A, subtypes 1/2. PFA*: Posterior fossa ependymoma group A (molecular group assigned based on RT-qPCR). PFB: Posterior fossa ependymoma group B. N/A: not available. Table provided as a separate file.

File Name: Supplementary Data 2
Description: **Differentially expressed genes in each cell of the cell populations identified in the single-nucleus RNA-seq data.** For each expressed gene, the log2(fold change) between the cell population and the rest of the cells and the adjusted p-value (p_{adj}) are presented. Fisher's method combined p-value, adjusted for multiple hypothesis testing using Benjamini-Hochberg procedure. Table provided as a separate file.

File Name: Supplementary Data 3
Description: **Significant ligand and receptor/co-receptor pair interactions and paracrine signaling pathways inferred by CellChat from the single-nucleus RNA-seq data.** The tabs "Signaling Pathways" and "Ligand-Receptor Interactions" respectively list the significant paracrine signaling pathways and individual paracrine interactions, together with their adjusted p-values (p_{adj}). 2-sided Wilcoxon rank-sum test p-value, adjusted for multiple hypothesis testing using Bonferroni procedure. Table provided as a separate file.

File Name: Supplementary Data 4
Description: **Differentially expressed genes between MLCs and neuroepithelial-like tumor cells in the single-nucleus RNA-seq data.** For each differentially expressed gene, the log2(fold change) between the cell population and the rest of the cells and the adjusted p-value (p_{adj}) are presented. Fisher's method combined p-value, adjusted for multiple hypothesis testing using Benjamini-Hochberg procedure. Table provided as a separate file

File Name: Supplementary Data 5
Description: **Transcription factors with differentially accessible binding motifs in each of the cell populations identified in the single-nucleus ATAC-seq data.** For each transcription factor, the JASPAR database motif id (Motif Id), the average transcription factor deviation score (z), the difference between average deviation scores in the cell population and the rest of the cells (Δz), and the adjusted p-value (p_{adj}) are presented. Fisher's method combined p-value, adjusted for multiple hypothesis testing using Benjamini-Hochberg procedure. Table provided as a separate file.

File Name: Supplementary Data 6
Description: **Differentially accessible peaks in each of the cell populations identified in the single-nucleus ATAC-seq data.** For each significant peak, the average accessibility log2(fold change) between the cell population and the rest of the cells, the adjusted p-value (p_{adj}), and the genes associated with the enhancer at that genomic locus according to the GeneHancer database are presented. Fisher's method combined p-value, adjusted for multiple hypothesis testing using Benjamini-Hochberg procedure. Table provided as a separate file.

File Name: Supplementary Data 7

Description: **Transcription factors with differentially accessible binding motifs between MLCs and neuroepithelial-like tumor cells in the single-nucleus ATAC-seq data.** The transcription factors that are differentially active in the MLC and in neuroepithelial cell populations according to the single-nucleus ATAC-seq data are presented in the tabs "MLC TFs" and "Neuroepithelial TFs", respectively. For each transcription factor, the JASPAR database motif id (Motif Id), the difference between the deviation scores in MLCs and neuroepithelial cells (Δz), the adjusted p-value (p_{adj}), the Spearman's correlation coefficient between the mRNA expression of the transcription factor and the abundance of MLCs in patients from the CBTN and Heidelberg cohorts (r_{CBTN} and $r_{Heidelberg}$), and their respective pvalues (pCBTN and pHeidelberg) are presented. Transcription factors with differentially accessible binding motifs and significant (p-value < 0.05) values for r_{CBTN} and $r_{Heidelberg}$ are marked in bold. Fisher's method combined p-value adjusted for multiple-hypothesis testing using BenjaminiHochberg procedure. Table provided as a separate file.

File Name: Supplementary Data 8
Description: **Genes with significant variability (Laplacian score) of expression within the MLC population in the single-nucleus RNA-seq data.** For each significant gene, the value of the Laplacian score (R_0) and the adjusted p-value (p_{adj}) are presented. Permutation test, p-value adjusted for multiple hypothesis testing using Benjamini-Hochberg procedure. Table provided as a separate file.

File Name: Supplementary Data 9
Description: **Differentially expressed genes in each of the MLC subpopulations identified in the single-nucleus RNA-seq data.** For each differentially expressed gene, the $\log_2$(fold change) between the cell population and the rest of the cells and the adjusted p-value (p_{adj}) computed with edgeR are presented. Table provided as a separate file.

File Name: Supplementary Data 10
Description: **Transcription factors with significant variability (Laplacian score) of binding motif accessibility within the MLC population in the single-nucleus ATAC-seq data. For** each significant transcription factor, the value of the Laplacian score (R_0) and the adjusted p-value (p_{adj}) are presented. Permutation test, p-value adjusted for multiple hypothesis testing using Benjamini-Hochberg procedure. Table provided as a separate file.
