# Supplementary material for: Pro-inflammatory cytokines mediate the epithelial-to-mesenchymal-like transition of pediatric posterior fossa ependymoma
Source: Nat Commun. 2022 Jul 8;13:3936. doi: 10.1038/s41467-022-31683-9 (PMC9270322; doi:10.1038/s41467-022-31683-9)
Supplement: Supplementary file 14 — Reporting Summary [file 41467_2022_31683_MOESM14_ESM.pdf]

nature portfolio

Corresponding author(s): Pablo G. Camara

Last updated by author(s): Jun 27, 2022

Reporting Summary

Nature Portfolio wishes to improve the reproducibility of the work that we publish. This form provides structure for consistency and transparency in reporting. For further information on Nature Portfolio policies, see our Editorial Policies and the Editorial Policy Checklist.

Statistics

For all statistical analyses, confirm that the following items are present in the figure legend, table legend, main text, or Methods section.

n/a	Confirmed	
☐	☒	The exact sample size (*n*) for each experimental group/condition, given as a discrete number and unit of measurement
☐	☒	A statement on whether measurements were taken from distinct samples or whether the same sample was measured repeatedly
☐	☒	The statistical test(s) used AND whether they are one- or two-sided<br>*Only common tests should be described solely by name; describe more complex techniques in the Methods section.*
☒	☐	A description of all covariates tested
☐	☒	A description of any assumptions or corrections, such as tests of normality and adjustment for multiple comparisons
☐	☒	A full description of the statistical parameters including central tendency (e.g. means) or other basic estimates (e.g. regression coefficient) AND variation (e.g. standard deviation) or associated estimates of uncertainty (e.g. confidence intervals)
☐	☒	For null hypothesis testing, the test statistic (e.g. *F*, *t*, *r*) with confidence intervals, effect sizes, degrees of freedom and *P* value noted<br>*Give P values as exact values whenever suitable.*
☒	☐	For Bayesian analysis, information on the choice of priors and Markov chain Monte Carlo settings
☒	☐	For hierarchical and complex designs, identification of the appropriate level for tests and full reporting of outcomes
☐	☒	Estimates of effect sizes (e.g. Cohen's *d*, Pearson's *r*), indicating how they were calculated

Our web collection on statistics for biologists contains articles on many of the points above.

Software and code

Policy information about availability of computer code

Data collection	No software was used for data collection.
Data analysis	Kallisto v0.45.1, EPN_Classifier (https://doi.org/10.5281/zenodo.6607426), Limma v3.34.1, STAR v2.6.1a, Drop-seq computational pipeline v2.3.0, Seurat v3.2.2, Harmony v0.1.0, Velocyto (https://github.com/velocyto-team/velocyto.R), scVelo 0.2.4, edgeR v3.20.1, CellChat v1.0.0, RayleighSelection (https://github.com/CamaraLab/RayleighSelection), msigdbr v7.0.1, fgsea v1.21.0, CIBERSORTx (https://cibersortx.stanford.edu/), Cell Ranger ATAC v2.0, Signac v1.1.0, GenomicRanges v1.38.0, Copy-scAT v0.3, chromVAR v1.8.0, ImageJ 2.35. See the methods section for details. The code used for the classification of ependymal tumors based on their gene expression is available at zenodo and the corresponding DOI is as follows: https://doi.org/10.5281/zenodo.6607426[99].

For manuscripts utilizing custom algorithms or software that are central to the research but not yet described in published literature, software must be made available to editors and reviewers. We strongly encourage code deposition in a community repository (e.g. GitHub). See the Nature Portfolio guidelines for submitting code & software for further information.

Data

Policy information about availability of data

All manuscripts must include a data availability statement. This statement should provide the following information, where applicable:

- Accession codes, unique identifiers, or web links for publicly available datasets
- A description of any restrictions on data availability
- For clinical datasets or third party data, please ensure that the statement adheres to our policy

The raw and processed single-nuclei RNA and ATAC-seq data generated in this study have been deposited in the Short Read Archive (SRA)/Gene Expression Omnibus (GEO) databases with accession number GSE206580 (https://www.ncbi.nlm.nih.gov/geo/query/acc.cgi?acc=GSE206580). The publicly available bulk RNA sequencing

data[25] used in this study are available from the Kids First Data Resource Portal (https://portal.kidsfirstdrc.org, project PBTA-CBTN). The publicly available gene expression data of the Heidelberg ependymoma cohort[5] used in this study are available from the GEO database with accession code GSE64415 (https://www.ncbi.nlm.nih.gov/geo/query/acc.cgi?acc=GSE64415). The JASPAR2020 database[98] used in this study is publicly available at https://jaspar.genereg.net/downloads/. The publicly available hallmark gene signatures[92] used in this study are available from the MSigDB database (https://www.gsea-msigdb.org/gsea/msigdb/collections.jsp#H). The mouse embryo RNA in situ hybridization data used in this study are available from the Allen Developing Mouse Brain Atlas (https://developingmouse.brain-map.org/). The GRCh38 human reference genome is available from Ensembl (http://ftp.ensembl.org/pub/release-106/fasta/homo_sapiens/dna/). Source data are provided with this paper. The remaining data are available within the Article, Supplementary Information, or Source Data file.

Field-specific reporting

Please select the one below that is the best fit for your research. If you are not sure, read the appropriate sections before making your selection.

☒ Life sciences ☐ Behavioural & social sciences ☐ Ecological, evolutionary & environmental sciences

For a reference copy of the document with all sections, see nature.com/documents/nr-reporting-summary-flat.pdf

Life sciences study design

All studies must disclose on these points even when the disclosure is negative.

Sample size	We profiled 9 flash-frozen human tumor samples with single-nuclei RNA-seq. We collected around 4,000 cells per sample. After quality control and filtration, a total of 25,349 cells with a mean of 544 expressed genes per cell were retained for analysis. We profiled 6 flash-frozen human tumor samples with single-nuclei ATAC-seq. We collected around 4,000 cells per sample. After quality control and filtration, a total of 14,461 cells and 229,286 accessible peaks were retained for analysis. These sample sizes were chosen based on sample availability, are comparable to those in other single-nuclei RNA-seq and ATAC-seq studies, and led to statistically significant and reproducible results in our analyses.
Data exclusions	For snRNA-seq data analysis, we filtered out cells with a low number of UMIs or a high proportion of expressed mitochondrially encoded genes. The distribution of cells in this 2D space was bimodal, and for each sample we used a linear cut in this space to segregate high-quality cells (high number of UMIs and low percentage of mitochondrial genes) from low-quality cells and debris (high percentage of mitochondrial genes and low number of UMIs). We excluded 2 clusters of cells from the scRNA-seq analysis which did not have any differentially expressed genes other than ribosomal and heat shock genes. For snATAC-seq data analysis, we removed low-quality cells with a low number and percentage of fragments in peaks, a low ratio of mono-nucleosomal to nucleosome-free fragments, a low transcription start site (TSS) enrichment score, a high percentage of mitochondrial fragments, or a low percentage of fragments overlapping targeted sites.
Replication	We considered at least 2 biological samples for each condition in all the omics experiments. We performed at least 3 technical replicates for each sample in the snRNA-seq experiments. Differential gene expression and chromatin accessibility analyses were performed independently for each sample and consistency was evaluated using Fisher's method for combining p-values. The inference of correlates between cell population abundances and signaling from bulk gene expression data was performed in two independent cohorts (denoted as CBTN and Heidelberg in the manuscript). We used at least 3 biological replicates x 3 technical replicates in all the qPCR, EdU, and cell migration experiments. All results were consistently reproduced across replicates.
Randomization	Samples were processed independently of their clinical characteristics. Each sample was processed on a separate day and the ordering was random.
Blinding	The investigators were blinded to the clinical characteristics of each sample.

Reporting for specific materials, systems and methods

We require information from authors about some types of materials, experimental systems and methods used in many studies. Here, indicate whether each material, system or method listed is relevant to your study. If you are not sure if a list item applies to your research, read the appropriate section before selecting a response.

Materials & experimental systems

n/a	Involved in the study	
☒	☐	Antibodies
☐	☒	Eukaryotic cell lines
☒	☐	Palaeontology and archaeology
☒	☐	Animals and other organisms
☐	☒	Human research participants
☒	☐	Clinical data
☒	☐	Dual use research of concern

Methods

n/a	Involved in the study	
☒	☐	ChIP-seq
☒	☐	Flow cytometry
☒	☐	MRI-based neuroimaging

Eukaryotic cell lines

Policy information about cell lines

Cell line source(s)	The cell line EPD-210FHTC was acquired from the Brain Tumor Resource Lab at Fred Hutchinson.
Authentication	We did not authenticate the EPD-210FHTC cell line as it is an early passage cell line that has been recently authenticated by the Brain Tumor Resource Lab, from which we obtained the cell line, using whole-genome methylation and gene expression profiling (https://hgserver1.amc.nl/r2/imi2/pdx/pdf/olson/EPD-210FHTC_P3.PDF).
Mycoplasma contamination	The cell line EPD-210FHTC was tested were negative for mycoplasma contamination using a PCR based method.
Commonly misidentified lines (See ICLAC register)	None of the commonly mis-identified cell lines was used in this study.

Human research participants

Policy information about studies involving human research participants

Population characteristics	The flash frozen tumor tissue samples used in this study were acquired from the CBTN tissue bio-repository (approved bio-specimen project #29). We selected samples from pediatric ependymoma patients (ages < 16 years old) of both sexes, with an initial tumor location in the posterior fossa. The anatomic location of the tumors and their diagnosis were obtained from the deidentified reports provided by the CBTN bio-repository. The molecular identity of the tumors was confirmed based on the expression of gene markers in bulk RNA-seq data or RT-qPCR. For recurrent and metastatic samples, we considered samples with a separation of at least 8 months from the previous surgery.
Recruitment	Tissue samples in the CBTN bio-repository are collected by the CBTN consortium from surgeries or biopsies and immediately flash-frozen using liquid nitrogen vapor. All specimens collected by the CBTN are considered a gift from our donors and their families. The repository includes samples from both sexes and diverse ethnicity. We considered samples for which the age at the time of diagnosis was < 16 years old, their location was in the posterior fossa (for primary tumors and recurrences) or were derived from a primary tumor located in the posterior fossa (for metastases), and their RNA was well preserved according to the bioanalyzer. We do not expect the presence of any major biases in the recruitment that could impact the results of this paper.
Ethics oversight	All procedures in this work were performed according to the institutional regulations of the University of Pennsylvania and the Children´s Hospital of Philadelphia (CHOP). The specimens and data used in this study were provided by the CBTN in a deidentified form according to the U.S. Department of Health and Human Services regulations and were not considered as Human Subjects Research by the Institutional Review Board of the University of Pennsylvania.

Note that full information on the approval of the study protocol must also be provided in the manuscript.
